# Consequences of the failure of equipartition for the *p*–*V* behavior of liquid water and the hydration free energy components of a small protein†

**DOI:** 10.1039/d4sc08437c

**Published:** 2025-03-28

**Authors:** Dilipkumar N. Asthagiri, Arjun Valiya Parambathu, Thomas L. Beck

**Affiliations:** a Oak Ridge National Laboratory One Bethel Valley Road Oak Ridge TN 37830 USA asthagiridn@ornl.gov; b Chemical and Biomolecular Engineering, University of Delaware Newark DE 19716 USA

## Abstract

Earlier we showed that in the molecular dynamics simulation of a rigid model of water it is necessary to use an integration time-step *δ*_t_ ≤ 0.5 fs to ensure equipartition between translational and rotational modes. Here we extend that study in the *NVT* ensemble to *NpT* conditions and to an aqueous protein. We study neat liquid water with the rigid, SPC/E model and the protein BBA (PDB ID: 1FME) solvated in the rigid, TIP3P model. We examine integration time-steps ranging from 0.5 fs to 4.0 fs for various thermostat plus barostat combinations. We find that a small *δ*_t_ is necessary to ensure consistent prediction of the simulation volume. Hydrogen mass repartitioning alleviates the problem somewhat, but is ineffective for the typical time-step used with this approach. The compressibility, a measure of volume fluctuations, and the dielectric constant, a measure of dipole moment fluctuations, are also seen to be sensitive to *δ*_t_. Using the mean volume estimated from the *NpT* simulation, we examine the electrostatic and van der Waals contribution to the hydration free energy of the protein in the *NVT* ensemble. These contributions are also sensitive to *δ*_t_. In going from *δ*_t_ = 2 fs to *δ*_t_ = 0.5 fs, the change in the net electrostatic plus van der Waals contribution to the hydration of BBA is already in excess of the folding free energy reported for this protein.

## Abstract 2

In MD simulations of aqueous systems using a rigid-body description for water molecules, a small time-step is needed to ensure consistent prediction of simulated properties, notably the *p*–*V* behavior and thermodynamics of hydration.

## Graphical abstract

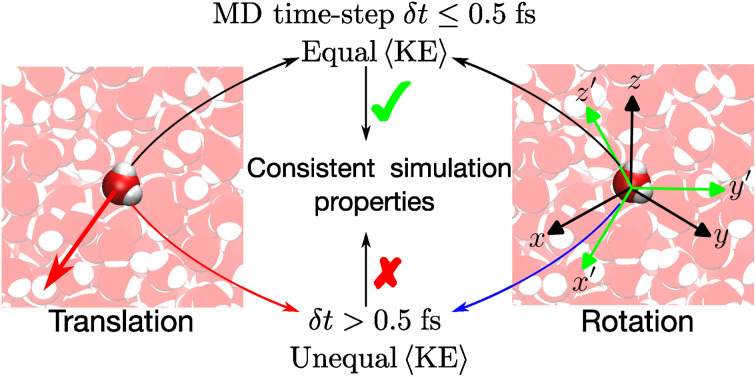

## 1 Introduction

Liquid water is the pre-eminent solvent for biological, geological, and chemical processes. Consistent with its pervasive role, it has been widely studied both experimentally and theoretically. In the theoretical and simulation context, modeling the intermolecular interactions and simulating liquid water has occupied a central place in the overall enterprise of computer simulation of materials.

Molecular dynamics simulations of water have a rich history. The seminal work by Rahman and Stillinger^1,2^ over half a century ago stands as a towering initial attempt to model the structure and dynamics of water. They described water as a rigid object, treating the translational motion using Cartesian coordinates and the rotational motion using Euler angles. Through careful analysis, they settled on a time-step *δ*_t_ = 0.4 fs for integrating the equations of motion. Some years later, Ryckaert, Ciccotti, and Berendsen introduced the SHAKE algorithm^3^ that enabled treating molecules such as water as a rigid object within a Cartesian coordinate system. SHAKE had the defect of not accounting for zero relative velocity of atoms connected by a rigid bond. This was fixed by the RATTLE algorithm.^4^ The subsequent development of the analytical SETTLE algorithm^5^ that obeys both the rigidity and velocity constraints for 3-site water molecules was another important innovation with significant impact in modeling water in bio-molecular simulations.

In molecular systems, the intra-molecular degrees are associated with stronger interaction energies or conversely higher frequency modes relative to the non-bonded, inter-molecular interactions. It is further the case that the higher frequency modes occur at time-scales that are well separated from the slower, inter-molecular dynamics. Thus, being able to freeze selected intra-molecular modes, as the constraint algorithms allow one to do, has the beneficial effect of removing from consideration modes that would otherwise require rather short time-steps to capture in a numerical scheme. Indeed, a key motivation in developing SHAKE, was the idea that “fast internal vibrations are usually decoupled from rotational and translational motions”.^3^ Thus, the thought was that if one could freeze the vibrational degrees of freedom, then one could take a longer time-step for describing the remaining slower rotational and translational motion. This reasoning has become central to modeling rigid-water molecular dynamics with time-steps that are considerably larger than the value used by Rahman and Stillinger. Most often, a value of 2 fs has been used in many studies over the last 4+ decades. It must then come as a surprise that recently we found that this assumption of decoupling of vibrations from rotations is not valid for water:^6^ the rotational relaxation in a fluid comprising rigid water molecules occurs on the same time-scales as the bond vibration and angle bending modes of water. (In water, the high frequency rotational motion is the librational motion in which the light hydrogen atoms wiggle about the axis passing through the heavier oxygen atom.) Thus, for water, taking a long time-step, assuming that “fast internal vibrations” have been frozen will still incur an error in describing the rotational relaxation, causing the breakdown of equipartition between translational and rotational motions. Ultimately, this breakdown is a consequence of not capturing both the translational and rotational relaxation with fidelity, as is required for obeying the fluctuation–dissipation relation for the respective modes.^7^ Our finding reconfirmed the breakdown of equipartition for bulk water noted in earlier works by Davidchack^8^ and Silveira and Abreu.^9,10^

For time-steps that lead to a breakdown of equipartition, we had found that the center of mass motion of water is at a higher temperature than the rotational motion about the center of mass. This suggested that for a constant volume simulation, the pressure must be higher for a larger *δ*_t_. Indeed, Davidchack had found exactly that behavior. We reasoned that under *NpT* conditions, the volume must be higher for a higher *δ*_t_. We validate this hypothesis in the present paper for both bulk water and for the designed protein with a ββα fold,^11^ hereafter termed protein BBA, solvated in water. For the bulk water simulation, the difference in volume between simulations with *δ*_t_ = 0.5 fs and *δ*_t_ = 2.0 fs can easily exceed typical volume changes in protein conformational change and protein unfolding. We also test the idea of hydrogen mass repartitioning,^12^ and find that while this helps somewhat, equipartition is still violated for the typical time-steps used with this approach.

We further reasoned that the isothermal compressibility and the static dielectric constant, properties that derive from the fluctuation of a related extensive quantity, should also depend on *δ*_t_. The calculation of these properties from the appropriate fluctuation relationship requires rather long simulations for adequate convergence (for example, see ref. 13). Nevertheless, the dependence of these fluctuation quantities on *δ*_t_ can still be inferred from the simulations. For exploring the dependence of the dielectric constant on *δ*_t_, we also study the electrostatic contribution to the free energy of hydration of a simple ion and a protein. For the simple ion, the charging free energy conforms to expectations based on the Born model and reveals the *δ*_t_-dependence of the dielectric constant. For the protein BBA in water, both the electrostatic and van der Waals (vdW) contribution to hydration depend on *δ*_t_. Importantly, in going from *δ*_t_ = 2.0 fs and *δ*_t_ = 0.5 fs, the change in the sum of the electrostatic and vdW contributions, an approximation to the net hydrophilic contribution in hydration, is comparable to the free energy of folding reported for BBA.^14,15^

## 2 Methodology

### 2.1 Bulk water

We studied the SPC/E^16^ water model using both NAMD^17,18^ and GPU-accelerated Tinker^19,20^ codes. The system comprised 4096 water molecules. Throughout, the equations of motion were integrated using the Velocity Verlet algorithm.

In NAMD, the Lennard-Jones and real-space electrostatic interactions were cutoff at 9 Å. Long-range, analytical LJ corrections were applied. The electrostatic interactions were calculated using particle mesh Ewald with a grid spacing of 1 Å. The relative Ewald energy tolerance at the real-space cutoff was 10^−7^, tighter than the default in NAMD. The system was first equilibrated for 6 ns under *NVT* conditions at a mass density of 1.014 gm per cc (≈1.5% higher than the value noted for SPC/E).^21^ We used the canonical stochastic velocity rescaling thermostat (CSVR)^22^ to maintain the system at 298.15 K and the time-step in this phase was 0.5 fs. The equilibrated end-point configuration was used as the starting configuration in all subsequent studies.

We next equilibrated the system under *NpT* conditions for 8 × 10^6^ steps followed by a production phase of 20 × 10^6^ steps. We simulated using time-steps ranging from 0.5 fs to 4.0 fs in 0.5 fs intervals. The geometry of the water molecule was maintained using the default SETTLE algorithm. Simulation data (energies, volumes, *etc.*) were archived every 500 steps for further analysis.

Within Tinker, we used the default Ewald cutoff of 7 Å and the default Lennard-Jones cutoff of 9 Å. Long-range LJ corrections were applied. The system was simulated for a total of 28 × 10^6^ steps as above, but data logging frequency changed with step size, being approximately every 1 ps (we say approximately since some time-steps do not evenly divide 1 ps). The geometry of water was maintained using the RATTLE algorithm. For simulations with Tinker, we also studied the effect of mass repartitioning^12^—the mass of the hydrogen atom in water was increased to 3.024 amu and the mass of oxygen appropriately reduced such that the mass of a water molecule remained at 18.0154 amu.

With NAMD, we experimented with the following thermostat-barostat combinations: Langevin thermostat/barostat,^23^ CSVR thermostat/Langevin barostat, and CSVR thermostat/Monte Carlo barostat.^24,25^ With Tinker, we used the Monte Carlo barostat and the CSVR thermostat. For the volume sampling frequency in the Monte Carlo barostat, we used the default values in the respective codes: 50 steps in NAMD and 25 steps in Tinker.

Within Tinker, for a limited set of runs, we experimented using the Beeman algorithm^26^ to learn if an improved estimate of on-step velocity affected the overall conclusions. These simulations were performed exclusively on CPUs.

Throughout, we use the Friedberg–Cameron approach^27,28^ to obtain statistical uncertainties for quantities such as the volume, the potential energy of the system, or the binding energy between a solute and the solvent (see below).

#### 2.1.1 Bulk reference

To provide a separate estimate of convergence of volume, we studied a larger 32 768 water system with a time-step of *δ*_t_ = 0.25 fs. This system was obtained by replicating the 4096 water system twice in the *x*, *y*, and *z* directions, respectively. The simulation box length was set to 100 Å; the bulk density was ≈2% lower than the converged value we found with the *δ*_t_ = 0.5 fs simulations. We equilibrated this system in the *NVT* ensemble for 6 × 10^6^ time-steps using the CSVR thermostat. The equilibrated configuration was then used to launch four separate *NpT* ensemble simulations using the CSVR thermostat and Monte Carlo barostat. The volume sampling frequency for the barostat was 80, 120, 160, and 200 time-steps, respectively, for the four separate runs. The *NpT* simulations were equilibrated for 6.25 × 10^6^ steps and data collected over an additional 6.25 × 10^6^ steps. In reporting the data for this larger system, the means from the four separate runs are averaged and the standard error of the mean obtained using variance propagation rules.

### 2.2 Aqueous BBA

BBA (PDB ID: 1FME) is a 28 residue designed protein that adopts a ββα fold. This is a marginally stable protein derived from a parent zinc-finger template^11^ sans the zinc ion. The first model from the PDB data file was taken and solvated in 6561 TIP3P^29^ water molecules. The N- and C-termini were modeled in the ammonium and carboxylate forms, respectively. At pH 7.0, the protein has a net charge of +4*e*, where *e* is the elementary charge; the net charge of the protein was compensated by adding 4 chloride (Cl^−^) ions to the system. The protein was modeled using the CHARMM36m^30–33^ forcefield (including CMAP corrections). CHARMM-modified parameters were used for TIP3P.^34^ The initial structure was built using the PSFGEN tool^35^ and the chloride ions were added using the autoionize tool within VMD.^36^

In the first set of simulations, no structural constraints were placed on the protein. The initial system was equilibrated under *NpT* conditions using *δ*_t_ = 0.5 fs for 4 ns (8 × 10^6^ steps). The Lennard-Jones forces were smoothly switched to zero from 9.43 Å to 10.43 Å. The particle mesh Ewald method was used for long-range electrostatic interactions, and as above, a tighter tolerance was used for Ewald summations. The bond between a hydrogen and the parent heavy atom was made rigid using the RIGIDBONDS ALL flag in NAMD. In this phase of equilibration, we used the Langevin thermostat and barostat.

The configuration from the end-point of the equilibration run was used to launch simulations at time-steps from 0.5 fs to 3.5 fs in intervals of 0.5 fs. For these studies we experimented with the following thermostat and barostat combinations: Langevin thermostat/barostat and CSVR thermostat/Monte Carlo barostat. For each *δ*_t_, the system was equilibrated over 12 × 10^6^ steps and data collected over a subsequent 20 × 10^6^ steps, with data logged every 500 steps. The simulation trajectory was archived every 1000 steps for further analysis.

#### 2.2.1 Hydration free energy components of BBA

For calculating the hydrophilic contributions to the hydration free energy of BBA, we made two important changes. First, we fixed the protein conformation. Second, we removed the Cl^−^ ions (within the Ewald formulation, the uniform compensating background charge ensures the electroneutrality of the system^37,38^). These changes were made to ensure that protein conformational fluctuations or variation in the binding of Cl^−^ ions to the protein do not obfuscate the role of *δ*_t_. The initial protein conformation was obtained by scanning the *NpT* simulation (using the CSVR thermostat/MC barostat and *δ*_t_ = 0.5 fs) for the conformer with the least deviation from the reference 1FME conformer. The RMS deviation of the chosen structure relative to the original 1FME structure was 1.55 Å.

Since we fix the conformation of the protein, we cannot use GPU-resident calculations within NAMD. (For the same reason, we cannot use the Monte Carlo barostat, as this is only available in the GPU-resident mode.) Thus, we used the CSVR thermostat and Langevin barostat to equilibrate the volume. Once the volume stabilized, we removed the barostat as well. The hydration free energy calculations were then performed in the *NVT* ensemble.

The electrostatic contribution to the free energy, *μ*^(ex)^_elec_, was obtained by a thermodynamic integration procedure using a three point Gauss–Legendre quadrature,^39,40^ with protein charges scaled by 
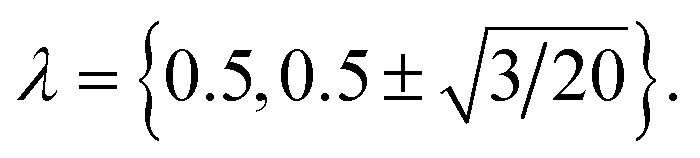
 Specifically,1
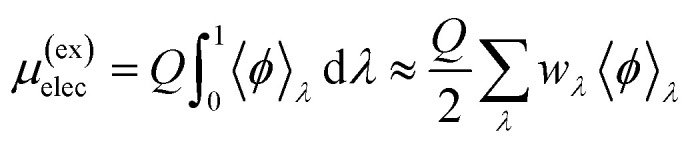
where 〈*ϕ*〉_*λ*_ is the electrostatic contribution to the interaction energy between the protein and the solvent with configurations sampled from the ensemble with charges scaled by *λ*; *w*_*λ*_ is the weight associated with the sampling point *λ*; and *Q* = +4*e* is the net charge of the protein. (N.B. *λ* = 0.5 gives the linear-response estimate of *μ*^(ex)^_elec_.) To complete the calculation, it is necessary to consider (Wigner) self-interaction^37,38^ and finite-size^41^ corrections. These scale with the length, *L*, of the cubic simulation box as 1/*L* and (*R*/*L*)^2^ × 1/*L*, respectively, where *R* is the nominal radius of the protein. Although the volume changes with *δ*_t_, as discussed below, the impact on the change in the Wigner self interaction contribution proves to be small, especially between *δ*_t_ = 0.5 fs and *δ*_t_ = 2.0 fs. Further, since the protein occupies a small volume of the simulation cell, we ignore finite-size corrections as well.

At each *λ* point, the system was equilibrated for 7.5 × 10^6^ steps and configurations archived every 500 steps in the subsequent production run of 7.5 × 10^6^ steps. The pair interaction approach in NAMD was used to calculate 〈*ϕ*〉_*λ*_.

The van der Waals (or nonpolar) contribution, *μ*^(ex)^_vdW_, to the free energy of hydration can be calculated from the quasichemical organization of the potential distribution theorem^42,43^ for a solute with all partial charges set to zero. Specifically,^44^

2 *μ*^(ex)^_vdW_ = *μ*^(ex)^_HC_ + *k*_B_*T* ln *p*(*n* = 0) + *k*_B_*T* ln∫*P*(*ε*|*n* = 0)*e*^*βε*^d*ε*,

where *μ*^(ex)^_HC_ is the hard-core (or packing) contribution to the hydration from a solute that simply excludes the solvent from a volume comprising the solute plus a defined inner shell; *P*(*ε*|*n* = 0) is the probability distribution of the binding energy of the solute with the solvent, subject to the inner-shell being bereft (*n* = 0) of solvent; *p*(*n* = 0) is the associated marginal distribution; and *β* = 1/*k*_B_*T* is the reciprocal temperature in energy units. A rigorous calculation of the terms in the above equation has been presented in the past,^44–48^ but such calculations are demanding and require supplying external forces to NAMD using the Tcl interface. In ref. 6 we have already established that for a small cavity, *μ*^(ex)^_HC_ is sensitive to *δ*_t_. Since our interest here is mainly to detect the role of *δ*_t_ in the protein–solvent interaction energy, we adopted the following procedure that will be accessible to most users of simulation codes. We completely ignored the excluded volume contribution and considered the molecular envelope as the inner shell, thus *p*(*n* = 0) = 1. We then computed the binding energy distribution, *P*(*ε*), of the protein with the solvent. If *P*(*ε*) is Gaussian with mean 〈*ε*〉 and variance *σ*^2^, and subject to the aforementioned simplifications, the vdW contribution, *μ*^(ex)^_vdW_, is given by^43,44^3
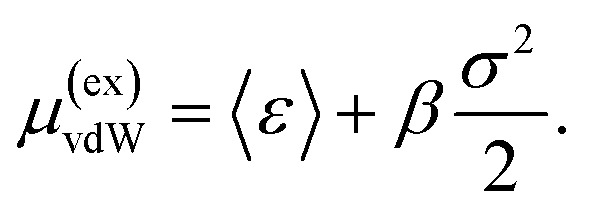
We find that the difference in *μ*^(ex)^_vdW_ between adjacent *δ*_t_ values is insensitive to whether or not we include the fluctuation contribution (*βσ*^2^/2). Hence, for further simplicity, we adopt the mean-field approximation *μ*^(ex)^_vdW_ ≈ 〈*ε*〉.

### 2.3 Fluctuation properties

The isothermal compressibility, *κ*_T_, is given by4
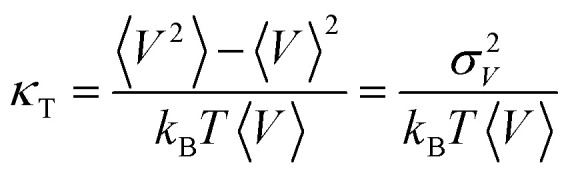
where 
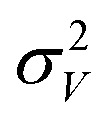
 is the second central moment of the distribution of *V* obtained in the simulations. If the sample of *V* obtained from the simulation is independent, identically distributed (iid), then we know that *σ*_*V*_ is *χ*^2^ distributed^49^ with the optimal value of *σ*_*V*_ given by 
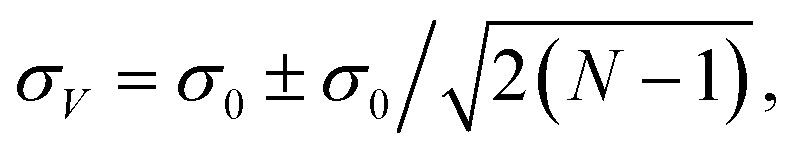
 where *σ*_0_ is the sample standard deviation of *V* and *N* is the sample size. However, the time-series trace of volumes in the simulation log is correlated. To this end, we compute the autocorrelation of *δ*_*V*_ = *V* − 〈*V*〉, and define the correlation length as the number of entries, *n*, in the log-file it takes for the normalized autocorrelation of *δ*_*V*_ to fall below 0.05. (An alternative choice for the correlation length is the statistical inefficiency from the Friedberg–Cameron approach; this choice is slightly tighter, but it still leads to the same mean *κ*_T_ and similar uncertainties.) We then sub-sample the time-series trace of *V*, such that the sampled *V* are separated by the auto-correlation length. For this sub-sample, we have 
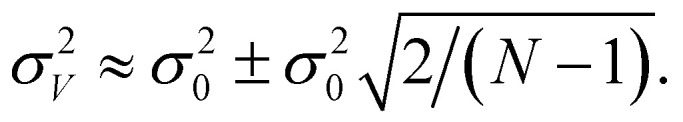
 (See also ref. 50.) By shifting the time origin, we construct *n* − 1 such sub-samples and compose the mean σ^2^_*V*_. The error of the mean *σ*^2^_*V*_ is then obtained using variance propagation rules.

In the *NVT* ensemble with conducting boundary conditions, the dielectric constant *ε* is given by^51^5
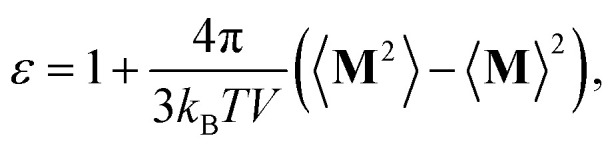
where **M** is the dipole moment of the simulation system; for an adequate sample size, 〈**M**〉 ≈ **0**. By expressing the equation in *x*, *y*, *z* components we follow the procedure used for calculating *κ*_T_ to calculate *ε* and its uncertainty. For calculating *ε* we used the average system size obtained using the MC barostat/CSVR thermostat with a time-step of 0.5 fs. We use the CSVR thermostat with 0.5 ps stochastic rescaling period and a production run of 50 × 10^6^ time-steps with configurations saved every 500 steps for analysis. We sought a second way to assess the impact of *δ*_t_ on the dielectric constant, namely by calculating the free energy of charging, *μ*^(ex)^_elec_, a Na atom^37^ to hypothetical charge states of +2*e* and +3*e*. The higher charge states help amplify the effects we are after. For these charging calculations, we add the ion to the simulation cell assuming the partial molar volume is zero, and rely on a linear-response approximation (*λ* = 0.5 noted above). The equilibration phase was for 5 × 10^6^ steps and the production phase lasted 40 × 10^6^ steps.

## 3 Results and discussion

### 3.1 Bulk water

**Fig. 1:**
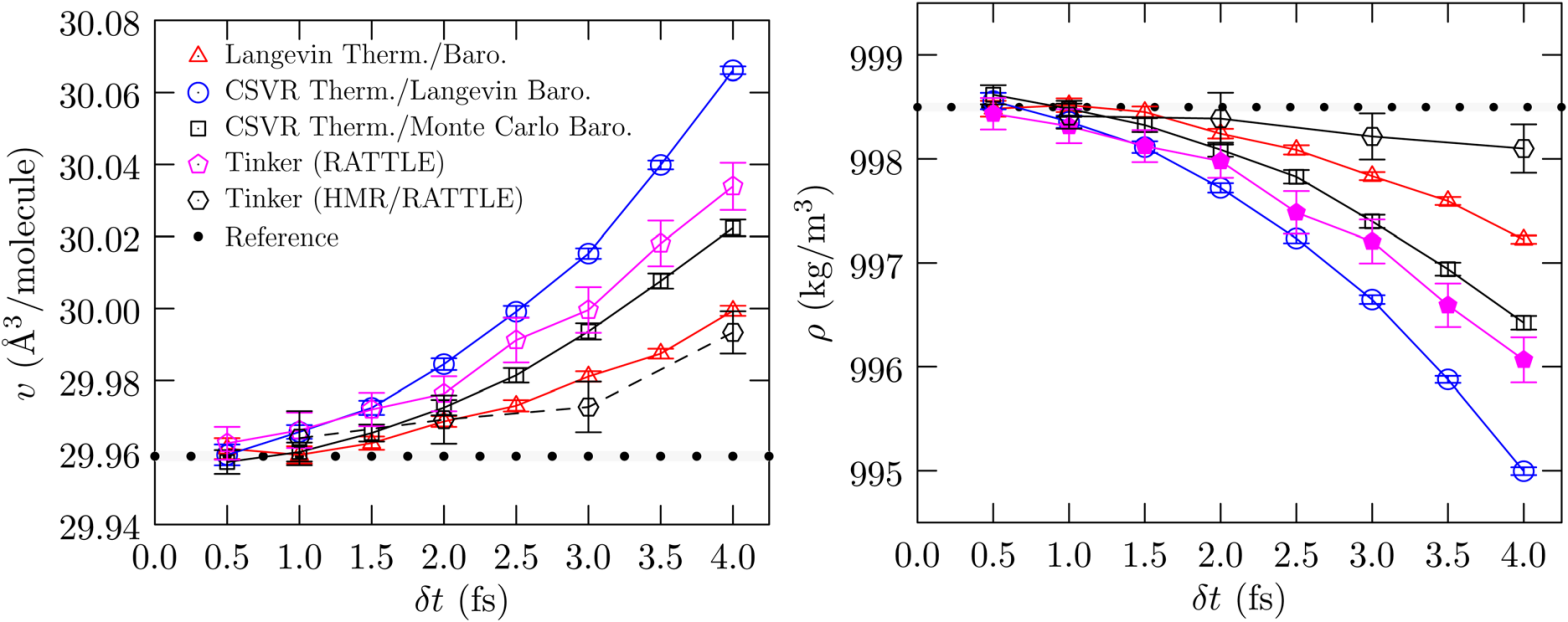
Water partial molar volume *v* (left panel) and mass density (right panel) *versus δ*_t_ for simulations with thermostat set at 298.15 K and barostat set at 1 bar. The simulations with Tinker use the CSVR thermostat and the Monte Carlo barostat. The standard error of the mean is shown at the 2*σ* level. Reference: 32 768 waters, *δ*_t_ = 0.25 fs. Data for different volume sampling frequencies are averaged and standard errors appropriately propagated. The radius of the symbol (•) is 2*σ* standard error of the mean.

Fig. 1 shows the volume (density) *versus δ*_t_ for different thermostat/barostat combinations. The horizontal line in the figure is the value obtained from averaging the four separate simulations for the large reference system. We find that by between 1250 steps, for a volume sampling frequency of 80, and 2500 steps, for a sampling frequency 200, the system volume settles close to the eventual mean value. This also helps confirm that the sample sizes for the reference calculation and the studies with the 4096 water system are rather conservative.

Fig. 1 shows that the volume increases with increasing *δ*_t_, irrespective of the thermostat/barostat combination. This is consistent with our hypothesis based on observations in the earlier study that the translational temperature increases as *δ*_t_ increases, and Davidchack's calculation of pressure *versus δ*_t_ in the *NVT* ensemble. Clearly, only for a small *δ*_t_—for the conditions tested here *δ*_t_ = 0.5 fs—the volumes (densities) converge to a common value that is independent of the thermostat/barostat combination. Importantly, the volumes (densities) converge to the value obtained from the entirely separate reference simulation. Lastly, hydrogen mass repartitioning (HMR) is an improvement in estimating the equilibrium volume (density), but the procedure is ineffective for *δ*_t_ = 4 fs. HMR can be defensible for *δ*_t_ ≤ 2.0 fs.

As a further check, we sought to compare with predictions of density from an entirely stochastic simulation. Sanz *et al.*^21^ have systematically explored the phase equilibrium of water for different water models using Monte Carlo calculations. For the SPC/E model and for a nearly identical treatment of inter-molecular interactions—they use a shorter cutoff of 8.5 Å *versus* 9 Å used by us—they quote a density of 1000 kg m^−3^ for 1 bar pressure and 300 K. Using the experimental thermal expansion coefficient for water, we can infer that at 298.15 K, the density from the Monte Carlo procedure should be about 1000.4 kg m^−3^. This value is an upper bound to the values obtained in the molecular dynamics simulations, with the least deviation of 0.2% of 998.5 kg m^−3^ obtained for *δ* = 0.5 fs. While this comparison is encouraging, we must note some caveats. First, Sanz *et al.* do not report the number of water molecules used in the simulations; we suspect^52^ it was considerably less than 4096, perhaps being as low as 360. As noted in our study on system size dependence of protein hydration,^53^ a larger system better accommodates density fluctuations and this may explain part of the deviation. (Exploring the relevance of this issue for converged density predictions is left for a separate study.) Second, Sanz *et al.* do not quote statistical uncertainties. We suspect^52^ it is about 1 kg m^−3^, in which case the agreement with our reference and *δ*_t_ = 0.5 fs results is satisfactory within the quoted statistical uncertainties of the respective simulations.

Consider next the change in the partial molar volume between *δ*_t_ = 0.5 fs and the more conventional *δ*_t_ = 2.0 fs. For simulations with the CSVR thermostat and Monte Carlo barostat, the partial molar volume for *δ*_t_ = 2.0 fs is about 0.02 Å^3^ larger. Thus for a system with 10 000 water molecules, a system size that is nowadays rather common and likely on the smaller size-scale of simulation systems, the volume for the *δ*_t_ = 2.0 fs simulation will be larger by about 200 Å^3^ relative to that for the system simulated with *δ*_t_ = 0.5 fs. To put this deviation in perspective, we note the following example. The volume change upon folding of the 149 residue Staphylococcal Nuclease protein at 21 °C, close to the temperature studied here, is found to be about 70 ml mol^−1^ or about 116 Å^3^ per molecule (of S. Nuclease).^54,55^ This deviation is already smaller than the error in overall system volume induced by too large of a *δ*_t_. Volume change upon folding/unfolding for similarly sized or smaller proteins will be comparable or smaller. Thus the impact of the artifacts due to too large a *δ*_t_ will be proportionally greater in assessing both the thermodynamics and the kinetics of the folding/unfolding transition in computer simulations.

**Fig. 2:**
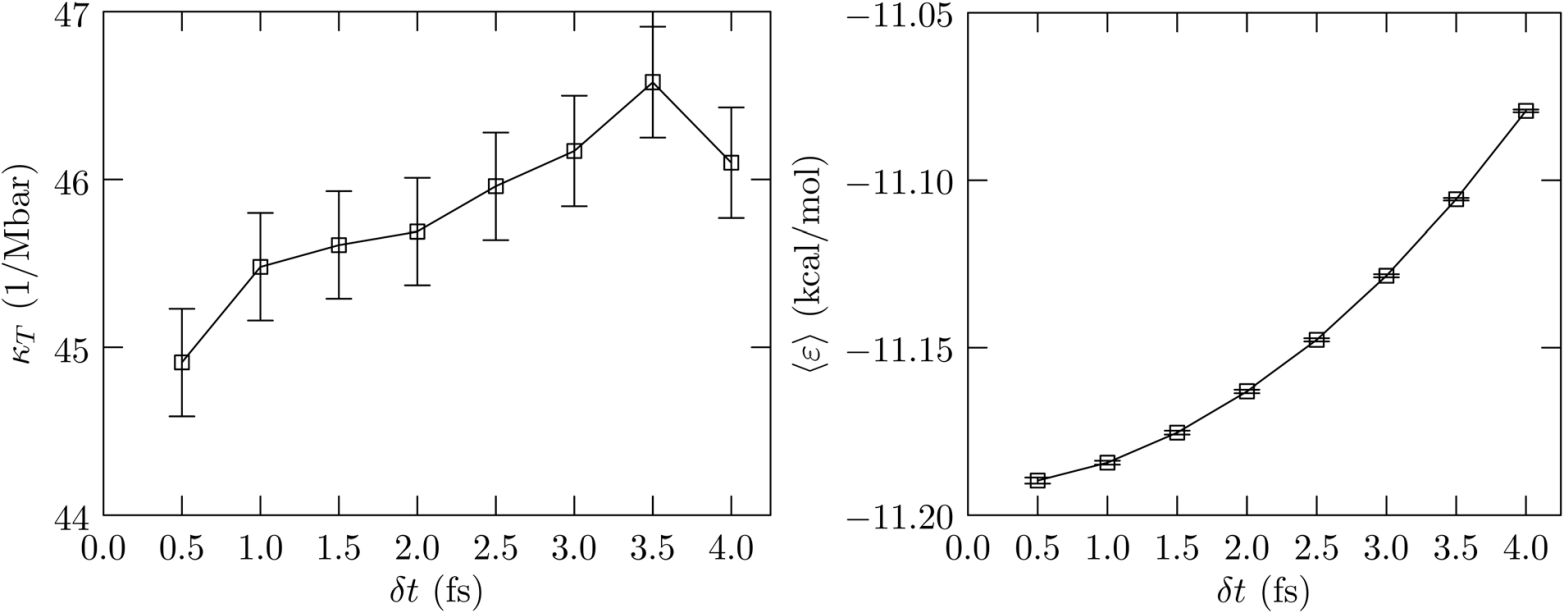
Left panel: Calculated compressibility *versus δ*_t_. The uncertainty is shown at the 1*σ* level. Right panel: The binding energy of a water molecule averaged over all frames. The standard error of the mean is shown at the 2*σ* level. The *NpT* calculations are performed with the CSVR thermostat and the Monte Carlo barostat within the NAMD program.

Since the volume depends on *δ*_t_, it begs the question whether the fluctuation in the volume under *NpT* conditions also depends on *δ*_t_. Fig. 2 shows the behavior of the estimated compressibility. For this analysis, we only consider the CSVR thermostat/Monte Carlo barostat; in contrast to the Langevin thermostat the CSVR thermostat is less intrusive in the dynamics and affects translation and rotation symmetrically. (With the CSVR thermostat the average of the translation and rotation temperatures equals the thermostat set-point temperature, unlike what we find for the Langevin thermostat.^6^)

In Fig. 2 (left panel), the estimated statistical uncertainties are large, as expected given the overall length of the simulation after accounting for correlations; however, the trend is unmistakable: the compressibility tends to decrease with decreasing *δ*_t_. The behavior of the compressibility with *δ*_t_ is also consistent with the behavior of the binding energy with *δ*_t_ (right panel): as cohesion increases one expects the fluid matrix to become stiffer and the compressibility to decrease.

**Fig. 3:**
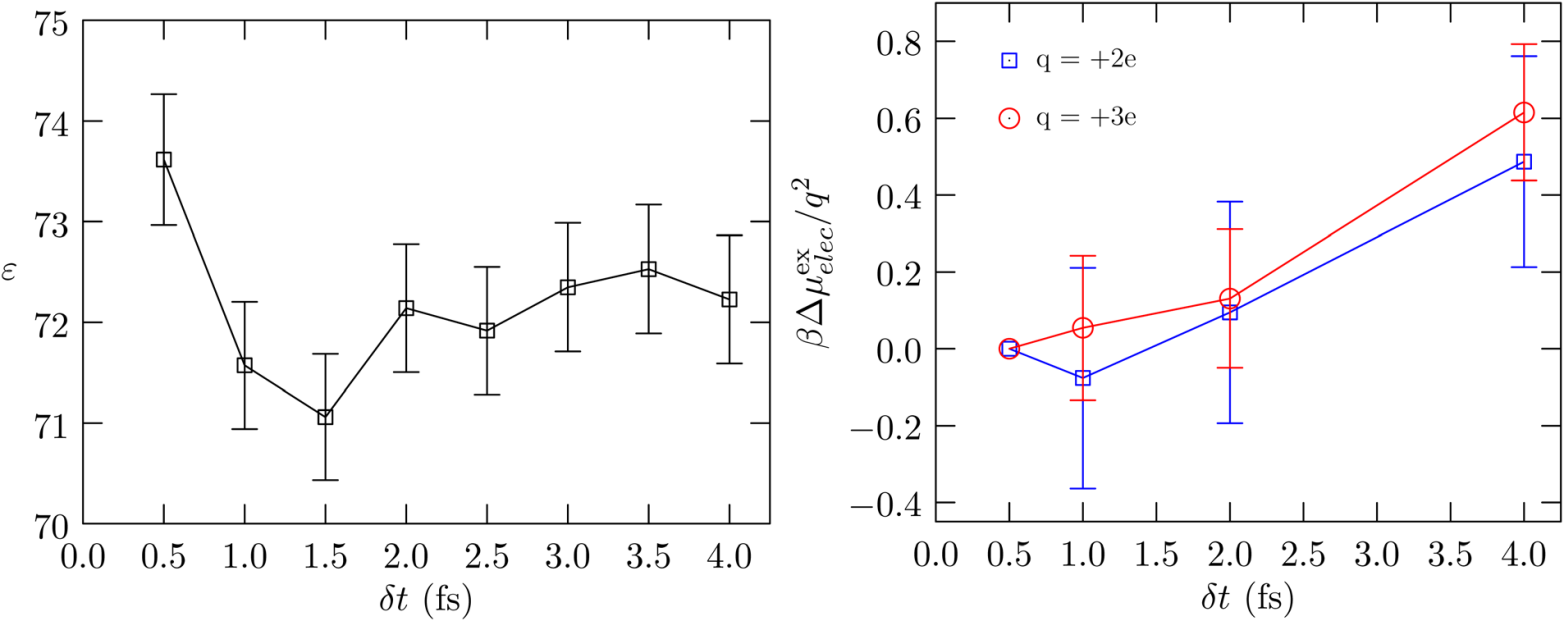
Left panel: Calculated dielectric constant *versus δ*_t_. The uncertainty is shown at the 1*σ* level. Right panel: The free energy of charging a Lennard-Jones Na^37^ atom to hypothetical charge states of *q* = 2*e* and *q* = 3*e*, respectively, relative to the corresponding value obtained using *δ*_t_ = 0.5 fs. The standard error of the mean is shown at the 1*σ* level.

Fig. 3 shows the calculated behavior of the dielectric constant and the behavior of β*μ*^(ex)^_elec_/*q*^2^. As seen in the calculation of *κ*_T_, the estimated statistical uncertainties in the calculation of *ε* are large. The data suggests that the dielectric constant can be sensitive to *δ*_t_, just as the compressibility is found to be sensitive to *δ*_t_, with the value for *δ*_t_ = 0.5 fs being slightly greater than the value for *δ*_t_ = 4.0 fs. This behavior is physically consistent with our earlier finding (Fig. 1, ref. 6) that as *δ*_t_ increases, the rotational motion is at a lower temperature relative to that for the translational motion. To better probe the electrostatic response of the fluid, we consider the problem of charging an ion (Fig. 3, right panel). Within the continuum dielectric Born model of hydration, β*μ*^(ex)^_elec_/*q*^2^ ∝ −(1 − 1/*ε*). However, with an atom that also has non-electrostatic interactions with the molecular fluid, to better isolate the dependence on the dielectric constant we consider the free energy of charging, βΔ*μ*^(ex)^_elec_/*q*^2^, relative to the value obtained using *δ*_t_ = 0.5 fs. The data shows that this difference quantity is roughly the same for both charge states, and consistent with our expectation, the dielectric constant is indicated to be higher for *δ*_t_ = 0.5 fs relative to *δ*_t_ = 4.0 fs. We find the same behavior in the more interesting case of the hydration of the protein BBA.

### 3.2 Aqueous BBA

**Fig. 4:**
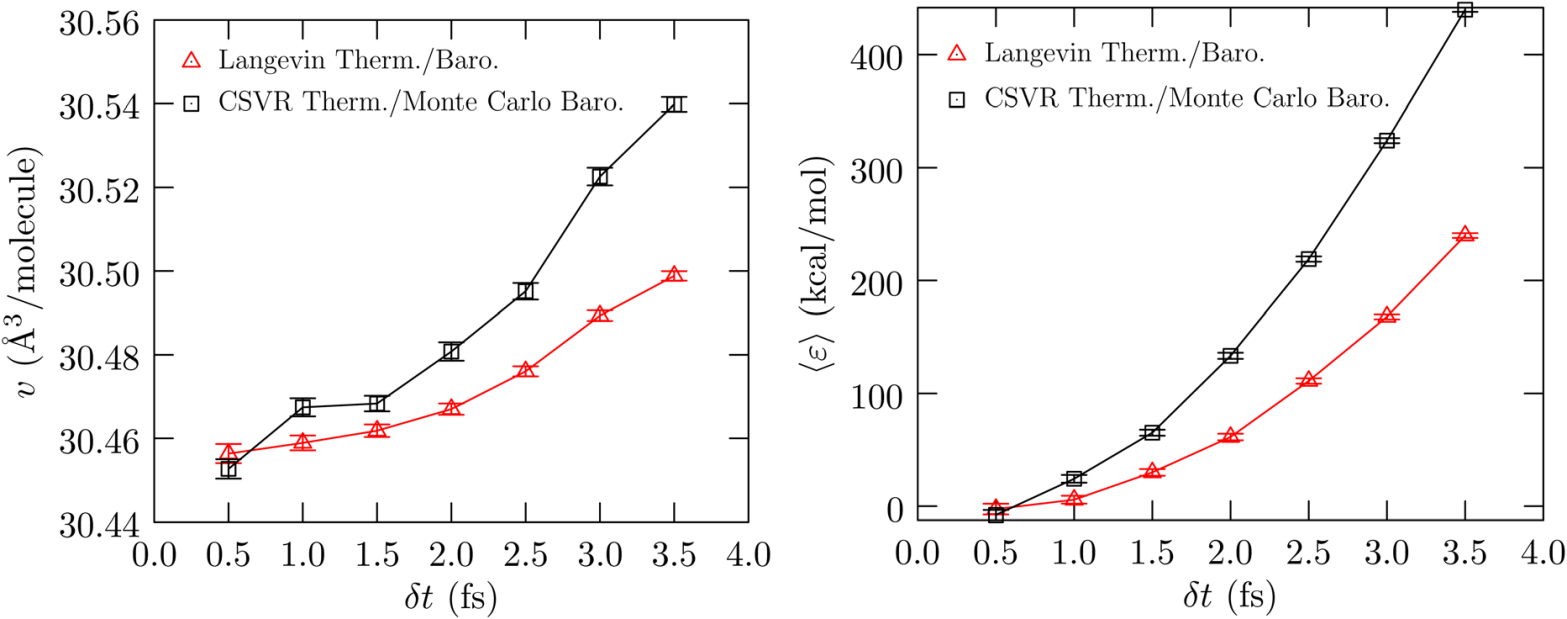
Left panel: Dependence of partial molar volume v of water in the presence of one molecule of BBA. Right panel: Total mean potential energy of the system relative to the value for *δ*_t_ = 0.5 fs. Thermostat is set at 298.15 K and barostat is set at 1 bar. The standard error of the mean is shown at the 2*σ* level.

Fig. 4 (left panel) shows the partial molar volume of water *versus δ*_t_ for the aqueous protein system. Just as we found for bulk water, the simulation volume converges to a value independent of the thermostat/barostat combination only for a small *δ*_t_. For the conditions tested here, *δ*_t_ = 0.5 fs ensures convergence of volume. It is heartening that this value of *δ*_t_ also ensure the convergence of the system potential energy to a common value.

Examining the dependence of the radius of gyration of the protein (*R*_g_) on *δ*_t_ in carefully constructed replica exchange simulations proved to be inconclusive in revealing systematic trends (data not shown). This can be due to confounding roles of flexibility, distribution of the counterion (Cl^−^), and the potential for artifacts introduced by violation of canonical sampling.^56^ To better isolate the dependence of protein–solvent interactions on *δ*_t_, we consider a protein in a fixed conformation in a water bath and without the counterion.

Table 1 shows the average equilibrium box length obtained from *NpT* calculations for this system. These values are used in the *NVT* ensemble calculations of *μ*^(ex)^_elec_ and *μ*^(ex)^_vdW_.

**Table 1:** Equilibrated volumes and the corresponding box length of the simulation with the protein in a fixed conformation

*δ*_t_ (fs)	*V* a (Å^3^)	*L* (Å)	*μ* ^(ex)^_self_ b (kcal mol^−1^)
0.5	200 074 ± 13	58.488	0.0
1.0	200 109 ± 9	58.491	0.0
2.0	200 289 ± 7	58.508	0.2
3.0	200 571 ± 6	58.536	0.4
4.0	200 978 ± 6	58.576	0.7

a Standard error of the mean at the 1*σ* level.

b The Wigner self-interaction correction^37,38^ is 
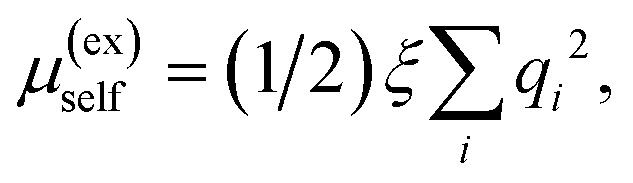
 where *q*_*i*_ is the partial charge of atom *i* of the protein and *ξ* = −2.837297/*L*. We report this correction relative to the *δ*_t_ = 0.5 fs case.

For the usual choice of *δ*_t_ = 2.0 fs relative to *δ*_t_ = 0.5 fs we can ignore the Wigner self-interaction correction for this system. Also, since the box is relatively large compared to the size of the protein, we ignore the finite-size correction to electrostatic interactions as well.

**Fig. 5:**
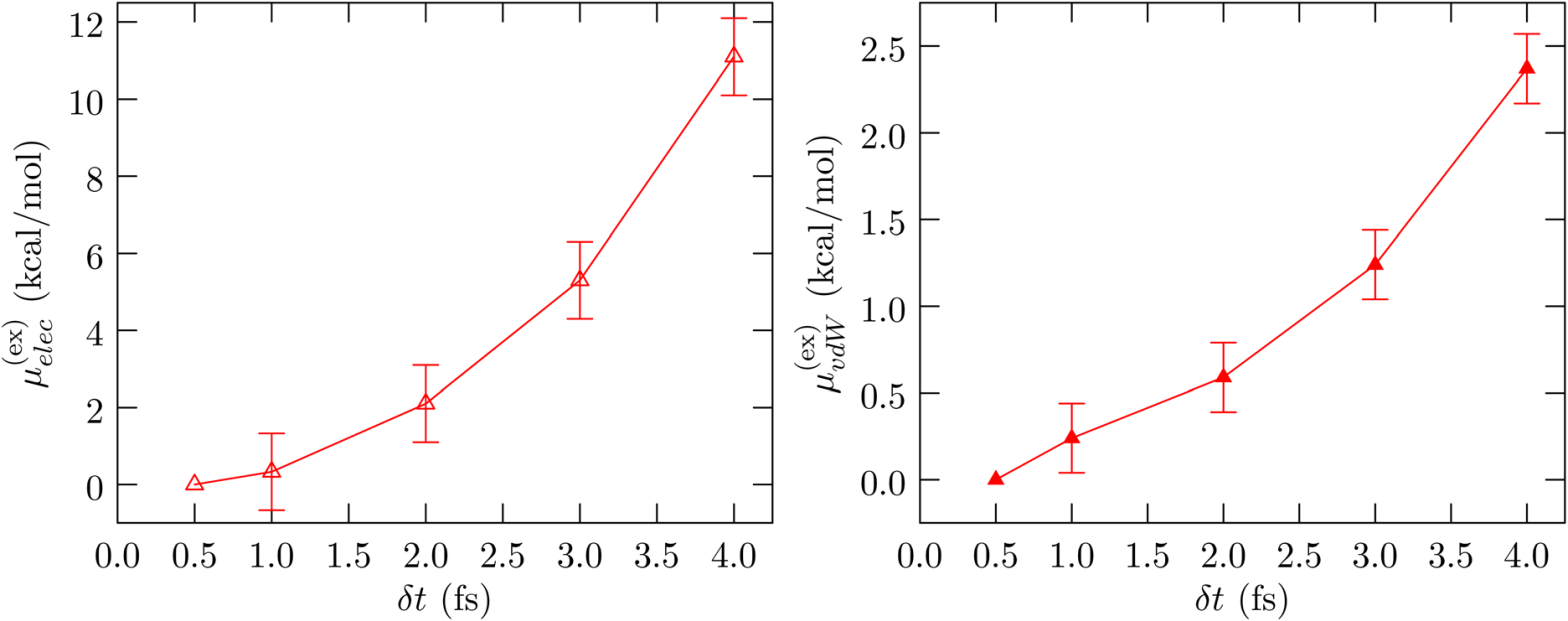
Left panel: Dependence of electrostatic contribution to the free energy of hydration on *δ*_t_. The Wigner self-interaction correction is not included. Right panel: *δ*_t_ dependence of mean-field van der Waals contribution to the hydration free energy. Throughout the numbers are shown relative to the mean value at *δ*_t_ = 0.5 fs. The standard error of the mean is shown at the 1*σ* level.

Fig. 5 shows *μ*^(ex)^_elec_ and *μ*^(ex)^_vdW_ of BBA. First, consider the electrostatic contribution. We find that the linear response result already gives ≈95% of the electrostatic contribution (data not shown), emphasizing that the three point rule is adequate in describing the charging free energy. Second, the behavior across *δ*_t_ shows that *μ*^(ex)^_elec_ is sensitive to *δ*_t_, even without including the Wigner self-interaction correction (Table 1) which amplify the trend even more. Finally, the vdW contribution obtained using the mean-field approximation *μ*^(ex)^_vdW_ ≈ 〈*ε*〉 is also sensitive to the step-size, although, as expected, not as strongly as *μ*^(ex)^_elec_.

Shaw and coworkers^15^ had studied the folding/unfolding transition of the BBA protein in very long computer simulations. At 325 K, they have calculated a folding free energy of about 0.7 kcal mol^−1^. (That study used a time-step of 2.5 fs.) Wu and Shea have estimated a similar value at 323 K.^14^ Assuming the folding free energy of the BBA protein is in a similar range at 298.15 K as well, we can see that the error introduced by a larger step-size in the calculation of the hydration free energy is already considerably larger than the reported folding free energy.

The thermodynamics of hydration and the conformational properties of biological macromolecules in liquid water are important quantities that inform the design and development of forcefields for biomolecular simulations. Our results suggest that tuning a forcefield in a simulation that does not obey equipartition is likely to introduce errors into the very structure of the forcefield itself. How these issues influence the kinetics and thermodynamics in aqueous and bio-macromolecular systems is necessarily left for future studies.

The earlier work^6^ and the present show that in a molecular dynamics approach to sampling equilibrium ensembles, it is important to capture the relevant relaxation dynamics with fidelity. However, in molecular dynamics sampling—and, for simplicity, we consider the NVE ensemble—the relevant metrics are conserved quantities, the foremost being the energy of the system; fidelity of capturing relaxation dynamics does not appear explicitly. In this regard, note that in discrete Hamiltonian dynamics with time-reversible algorithms, the sampling is from a shadow Hamiltonian, *H̃*(*δ*_t_) = *H* + *G*(*δ*_t_^2^), where *H* is the physical Hamiltonian.^57–60^ (The general form of *G* is unknown but is usually investigated by means of a series expansion.^61^) As a consequence, and within time-reversible algorithms, *H* is not conserved but oscillates about a mean value, with the size of oscillations proportional to *δ*_t_^2^.^60^ This discretization error, or what can also be thought of as an exchange of “shadow” work with the system,^62^ is what impacts the relaxation. Thus, we see that an algorithm can be globally stable with a long *δ*_t_, but that stability is not sufficient to ensure relaxation processes are correctly captured.

A further consequence of discrete Hamiltonian dynamics is that the particle velocity *v* ≠ *p*/*m*, where *p* is the momentum obtained using the “Velocity”-Verlet equation^59^ and *m* is its mass. Better estimates of *v* can be obtained and the temperature defined using the generalized equipartition theorem.^63,64^ This is an important point to consider. However, the key insight from the earlier work and the present one is the need to obey the fluctuation–dissipation theorem, *i.e.* the need to capture the temporal evolution of fluctuations^7^ or equivalently the underlying relaxation dynamics with fidelity in a MD sampling of ensembles.

For a molecular dynamics sampling of equilibrium ensembles, we suggest it would be prudent to study relevant velocity autocorrelations,^6^ or if that is too tedious, estimate quantities such as mean energy or volume for several different time-steps to ensure that time-step artifacts are under control. Such checks would be especially prudent before undertaking large-scale simulation campaigns.

## 4 Conclusions

It has been nearly 50 years since Ryckaert, Ciccotti, and Berendsen introduced the SHAKE algorithm that allowed the efficient description of molecules such as water as a rigid object in computer simulations. A guiding idea in this development was the assumption that fast internal vibrations in the molecule are decoupled from translational and vibrational modes, and by describing the molecule as a rigid object, it is permissible to take a longer time-step to integrate the equations of motion. However, this chain of reasoning is not valid for water. In particular, in the simulated dynamics of rigid water molecules the rotational relaxation occurs on time-scales that are comparable to the internal vibrations in the physical water molecule. Ignoring this fact and simulating a liquid comprising rigid water molecules with a long time-will lead to an incorrect description of the rotational relaxation.

The rapid angular motion in the dynamics of a fluid comprising rigid water molecules leads to the rotational relaxation occurring at a shorter time scale than the translation relaxation. This imposes a fundamental limit on how long a time-step one can use for correctly capturing the temporal evolution of fluctuations, as is required for adhering to the fluctuation–dissipation relation. A long time-step for integrating the equations of motion leads to an incorrect description of the temporal evolution of fluctuations in turn leading to the breakdown of equipartition. We find that for integrating the equations of motion for the rigid SPC/E and TIP3P models of water, a time-step of 0.5 fs is defensible, a value that is also close to what Rahman and Stillinger had suggested long ago from their analysis of the dynamics of a pair of water molecules.

The breakdown of equipartition leads to the translational modes being hotter than the rotational modes, a deviation that is amplified as the time-step is increased. As a consequence, at constant pressure, the volume is larger for a longer time-step. For a protein dissolved in water, the deviation in the partial molar volume will necessarily introduce uncontrolled *p*–*V* errors in the folding free energy landscape.

The *δ*_t_ artifact also impacts the interaction between the protein and solvent. For the BBA protein, the error introduced in the electrostatic plus vdW contribution to the hydration free energy can easily exceed the folding free energy. Thus, too long a time-step introduces errors in the interaction contribution, further obfuscating the resolution of the free energy landscape.

## Data availability

ASCII formatted simulation metadata and log files for all cases and the binary trajectory for BBA in water with *δ*_t_ = 0.5 fs (MC barostat) are available at DOI: 10.13139/OLCF/2480346. The data is also findable at doi.ccs.ornl.gov. The repository has a README.txt that can help in navigating the data.

## Author contributions

D. N. A.—conceptualization, simulation, data analysis, and writing; A. V. P.—simulation (with Beeman algorithm), manuscript review; T. L. B.—research oversight, manuscript editing.

## Conflicts of interest

There are no conflicts to declare.
